# A Novel and Non-Invasive Approach to Evaluating Soil Moisture without Soil Disturbances: Contactless Ultrasonic System

**DOI:** 10.3390/s22197450

**Published:** 2022-09-30

**Authors:** Dong Kook Woo, Wonseok Do, Jinyoung Hong, Hajin Choi

**Affiliations:** 1 Department of Civil Engineering, Keimyung University, Daegu 42601, Korea; 2 School of Architecture, Soongsil University, Seoul 06978, Korea

**Keywords:** Rayleigh wave, sensor, soil moisture, random forest

## Abstract

Soil moisture has been considered a key variable in governing the terrestrial ecosystem. However, it is challenging to preserve indigenous soil characteristics using conventional soil moisture monitoring methods that require maximum soil contacts. To overcome this issue, we developed a non-destructive method of evaluating soil moisture using a contactless ultrasonic system. This system was designed to measure leaky Rayleigh waves at the air–soil joint-half space. The influences of soil moisture on leaky Rayleigh waves were explored under sand, silt, and clay in a controlled experimental design. Our results showed that there were strong relationships between the energy and amplitude of leaky Rayleigh waves and soil moisture for all three soil cases. These results can be explained by reduced soil strengths during evaporation processes for coarse soil particles as opposed to fine soil particles. To evaluate soil moisture based on the dynamic parameters and wave properties obtained from the observed leaky Rayleigh waves, we used the random forest model. The accuracy of predicted soil moisture was exceptional for test data sets under all soil types (R${}^{2}$ ≥ 0.98, RMSE ≤ 0.0089 m${}^{3}$ m${}^{-3}$). That is, our study demonstrated that the leaky Rayleigh waves had great potential to continuously assess soil moisture variations without soil disturbances.

## 1. Introduction

It has long been recognized that soil moisture is a key variable in terrestrial ecosystems. Despite the amount of water held by the soil being a small fraction in the Earth’s system when compared with other hydrological components, the accurate estimates of soil moisture are of great importance to hydrological, agricultural, biogeochemical, and climate processes [1,2,3]. Soil moisture is a critical component of hydrological dynamics that governs surface and subsurface flow, infiltration, and evapotranspiration processes through the exchanges of surface energy fluxes [4,5]. The spatiotemporal variation of soil moisture is a key index to determining the timing and amount of crop irrigation [6,7]. Soil moisture plays a pivotal role in structuring soil microbial communities and affects the processes of organic matter decomposition, inorganic transformation, transport, and nutrient assimilation [8,9,10]. Carbon dioxide (CO${}_{2}$) emission from the soil can be also explained by the variations of soil moisture and temperature (that is a function of soil moisture as well) up to 90% [11] and is approximately 10 to 15 times greater than that from the burning of fossil fuels [12]. That is, it is imperative to have an accurate measure of soil moisture to address these intertwined dynamics.

There have been various techniques developed to estimate soil moisture for laboratory and in situ experiments. Classical methods are to use thermo-gravimetric analysis (oven drying) and calcium carbide reaction with water in the soil [13,14]. Although both methods are relatively simple and rapid, it is not practical to capture the temporal evolution of soil moisture [14,15]. In modern methods, the soil electrical property and soil water potential are used as proxies to monitor soil moisture [15,16]. These modern methods have improved the reliability of soil moisture estimations [14,17]. However, it is found that the installations of modern sensors are not straightforward since they should be completely immersed into the soil to ensure the accuracy of soil moisture readings [15]. In other words, it is difficult to minimize soil disturbances to preserve the original soil characteristics and structures around the sensors installed.

Several efforts have been made for addressing the major two issues described above (i.e., continuous and non-destructive measurements of soil moisture). One of the techniques to overcome these shortcomings is to utilize radioactive techniques, such as neutron scattering and gamma attenuation [18,19]. Ground penetration radar (GPR) has been also used to monitor soil moisture using the relative dielectric permittivity and electrical conductivity of the soil [20,21,22]. A study employed time-lapse GPR measurements to estimate soil moisture with high accuracy and repeatability [23]. However, compared with other methods, their ease of use, safety, and acceptability are still in question, and thus they will most likely not be widely adopted in the near future [15,24]. For an effective technique to monitor soil moisture in the top layer for both laboratory and in situ experiments, it should be inexpensive, safe, and easy to handle in combination with overcoming the major two issues [25]. In this context, a continuous elastic wave is an alternative option to monitor near-surface soil moisture dynamics. The elastic properties of sound propagation in porous media have been utilized to characterize soil moisture since the velocity of elastic waves may be dependent on soil porosity, density, and water content [26,27,28]. However, none of the previous studies satisfy all of these requirements, especially for preserving the original soil structure and properties.

The air-coupled ultrasonic method has been introduced as a promising non-destructive technique, where the testing configuration is fully contactless over the object [29]. The ultrasonic excitation and measurement are performed in the air, while the obtained elastic waves are mainly governed by the characteristics of solids [30,31]. Therefore, additional efforts for embedding or coupling sensors at specimens are not necessary. This technique measures leaky surface waves, propagating at the interface between the air and solid. In a joint-half space such as a fluid–solid case, the Rayleigh wave dominantly propagates along the surface of the solid, and a portion of the Rayleigh waves is leaked into the fluid. The leaky Rayleigh waves have a great benefit of near-surface material characterization without the disturbances of sensor installations. Due to the uniqueness of the testing scheme, the air-coupled ultrasonic method is employed in the characterization and non-destructive evaluation of industrial materials, such as paper, wood, and advanced composites [29,32]. In particular, recent studies demonstrated that the propagation of leaky Rayleigh waves is sensitive to phase change of cementitious materials [33,34]. Likewise, this technique could be used to assess the spatiotemporal variations of soil moisture without soil disturbances. That is, exploring the potential to use leaky Rayleigh waves to monitor soil moisture is the focus of this study to overcome the two key issues discussed above.

## 2. Method

**Figure 1:**
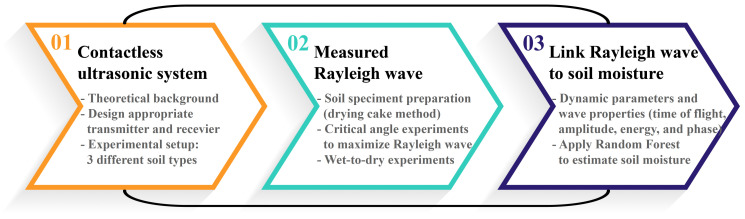
Overview of the proposed contactless ultrasonic method used in this study to estimate soil moisture.

An overview of the proposed nondestructive technique to estimate soil moisture using leaky Rayleigh waves is illustrated in Figure 1. The proposed approach is an alternative to conventional soil moisture measurement techniques to minimize soil disturbances. The contactless ultrasonic system developed in this study to measure leaky Rayleigh waves and estimate soil moisture contents is described below.

### 2.1. Theoretical Background of Rayleigh Wave

In a joint-half space, such as fluid and solid, a portion of the Rayleigh waves in the solid is leaked into the fluid, called a leaky Rayleigh wave [35]. The leaky waves are of great interest in the field of non-destructive testing because the characteristics of the solid can be identified by measuring the leakage in the fluid side. The characteristic equation of leaky Rayleigh wave can be presented as Equation (1):

(1) $$4k^{2}qs-{\left(k^{2}+s^{2}\right)}^{2}=i\frac{\rho_{L}}{\rho_{s}}\frac{qk_{t}^{4}}{\sqrt{k_{L}^{2}-k^{2}}}$$

where $\rho_{L}$ and $\rho_{S}$ are the densities of the fluid and solid, $k_{t}$ is the transverse wavenumber in the solid, $k_{L}$ is the wavenumber in the fluid, *k* is a wavenumber of the Rayleigh wave, and *q* and *s* are the longitudinal and transverse vertical wavenumbers, respectively. As the density ratio, $\rho_{L}/\rho_{S}$ becomes smaller, the wavenumber of the Rayleigh wave becomes a complex root and the Rayleigh wave radiates its energy into the fluid, forming a leaky Rayleigh wave. In the case of the air–soil joint-half space, the density ratio is less than 6.5 × 10${}^{-4}$, where the values of 1.3 kg m${}^{-3}$ and 2000 kg m${}^{-3}$ are used as representative of $\rho_{L}$ and $\rho_{S}$, respectively. In such a range of the density ratio, the characteristics of the leaky Rayleigh wave are almost identical to those of the Rayleigh wave [35].

The Rayleigh wave is a mechanical wave that propagates along the surface of a medium. The wave motion is elliptical, including a portion of P- and S-waves, where the dilatational and distortional motion is guided at the surface boundary. Therefore, the propagation of the Rayleigh wave is governed by characteristics of both P- and S-waves. In particular, the distortional motion is the unique behavior of the solid due to shear resistance. Recent studies show that the degree of Rayleigh wave propagation well correlates with the phase change of cementitious materials [33,34]. This is because the distortional motion becomes dominant depending on the degree of solidification in the medium. In this regard, the water content in the soil could be evaluated by Rayleigh wave propagations. It is hypothesized that the degree of soil hardness dependent on soil moisture causes the lack of distortional Rayleigh wave motion. As the soil moisture increases, the hardness of the soil decreases, and thus the amplitude of the Rayleigh wave is attenuated. When the soil moisture becomes fully saturated to disturb the wave motion, the Rayleigh wave does not propagate through the medium.

**Figure 2:**
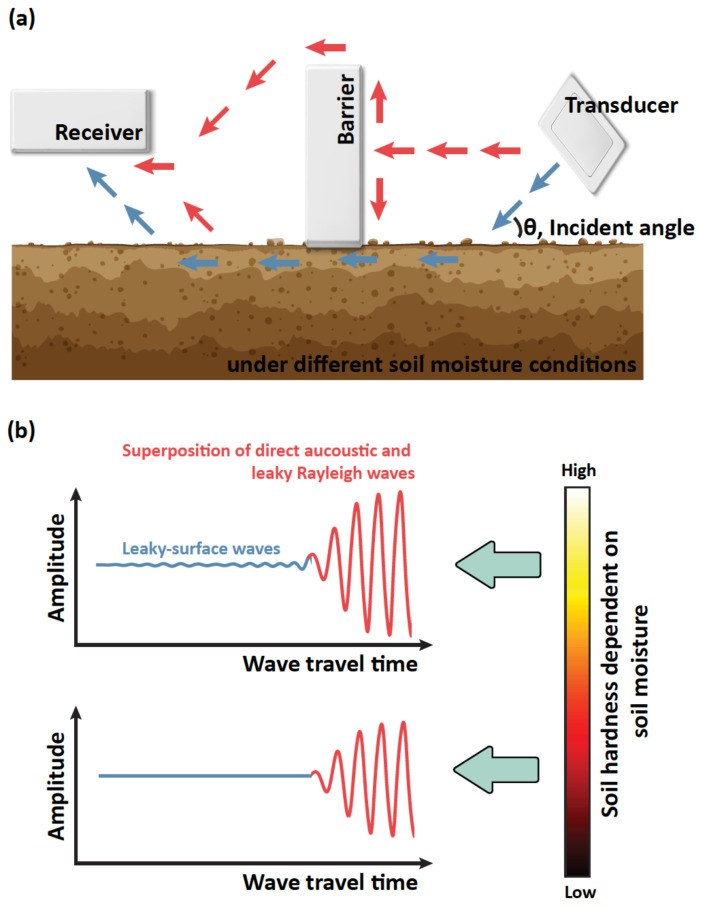
Schematic illustration of air-coupled ultrasonic method. (**a**) Propagation of leaky Rayleigh wave (blue) and direct acoustic (red). (**b**) Measurement scenario of leaky Rayleigh wave depending on soil moisture.

To measure the leakage of the Rayleigh wave propagating at the interface between the air and soil, an air-coupled testing configuration is required. The test set-up includes a contactless ultrasonic transducer, receiver, and acoustic barrier as described in Figure 2a. When an ultrasonic pulse is excited with an incident angle, the acoustic wave radiates from the transducer. The incident wave into the soil is converted to elastic waves, where the main portion of the energy is focused on the Rayleigh wave at the surface of the soil. It is noted that the incident angle is a key parameter to excite the maximum energy of the Rayleigh wave based on Snell’s law. As the Rayleigh wave propagates along the joint space, the leakage can be measured by a contactless receiver. The other waves radiating from the transducer propagate through the air, called the direct acoustic. The amplitude of the direct acoustic is considerably higher than that of the leaky Rayleigh wave, while the wave speed is slower. Therefore, the acoustic barrier is needed to delay and attenuate the direct acoustic in order to obtain more information of leaky Rayleigh waves. As illustrated in Figure 2b, the measured signal by the receiver is the superposition of the leaky Rayleigh wave and the direct acoustic. The characteristics of leaky Rayleigh waves would vary depending on the soil moisture; however, the direct acoustic is constant.

### 2.2. Contactless Ultrasonic System

**Figure 3:**
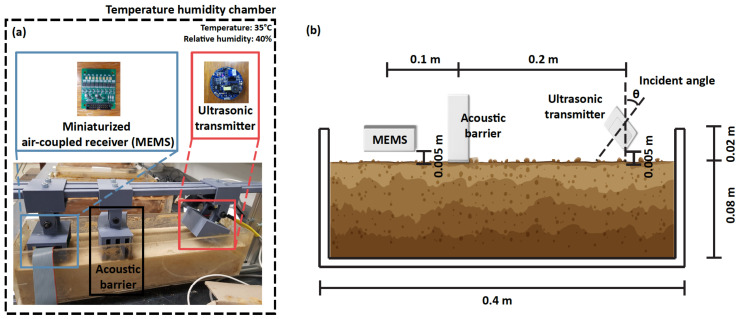
Test set-up. (**a**) Air-coupled ultrasonic receiver (left) and transducer (right). (**b**) Schematic diagram of the test set-up including the dimensions of specimens and distances between sensors, barrier, and soils.

The contactless ultrasonic system was carefully designed to measure leaky Rayleigh waves without touching the surface of the soil. As shown in Figure 3a, the developed system included an ultrasonic transmitter and receivers. The ultrasonic transmitter (PID-615089, SensComp, Livonia, MI, USA) generated 16 cycles of a sinusoidal waveform with a maximum peak of 200 voltages. The transmitter was an electrostatic ultrasonic transducer and the circular shape of the transducer resonated with the center frequency of 49.4 kHz when it was charged. The controlled electrical circuit was located in the backside of the transmitter, where the trigger of pulsation was obtained every 200 ms (5 Hz as pulse repetition rate). The receivers (SPU0410LR5H-QB, Knowles Acoustics, Japan) consisted of 8 micro-electromechanical systems (MEMS), where the sensors were located with 5 mm spacing in a row. The 8-channel microphone array was used for a robust and reliable data acquisition [34]. The aligned sensors measured the air pressure and the designed circuit amplified the obtained signals about 2000 times. We measured 3000 samples at a sampling rate of 2 MH and used NI-DAQ 6366 to digitize obtained signals.

The transmitter and receivers were attached in a 3D-printed frame and positioned apart from the soil specimen without disturbances. An acoustic barrier was placed between the transmitter and receivers. The barrier was composed of several layers of plastic 3D-printed materials and an air gap, which caused the delay and attenuation of the direct waves due to the acoustic mismatch [36]. That is, the acoustic barrier was used to ensure the excitement of Rayleigh waves because all-acoustic waves should be transmitted through the soil surface [37] and to compensate for small differences between the speed of sound in the air (343 m s${}^{-1}$) and the soil (355 to 1907 m s${}^{-1}$, Table 1).

### 2.3. Experimental Setup

Three primary soil types (sand, silt, and clay) were used in this study as presented in Table 1. The particle sizes of sand, silt, and clay were 2.0 to 0.05 mm, 0.05 to 0.002 mm, and less than 0.002 mm, respectively, and were classified based on the soil texture classifications defined by the United States Department of Agriculture [38]. Soil particle size distribution is a fundamental soil physical property. The soil texture and characteristics, including water-holding capacity, hydraulic conductivity, porosity, bulk density, aggregates, Poisson’s ratio, and modulus of elasticity, can be classified by the percentage of sand, silt, and clay (e.g., [39,40,41,42]). That is, distinct and diverse soil physical properties were considered to embrace a broad range of Rayleigh waves generated by different soil moisture and types.

The experimental design for the contactless ultrasonic transmitter and receiver to estimate soil moisture based on leaky Rayleigh waves was presented in Figure 3b. The specimen of the soil was placed in an acrylic box whose dimensions were 0.4 m (length) × 0.1 m (width) × 0.08 m (height). The ultrasonic transmitter and receiver were positioned approximately 0.005 m (lift-off distance) from the soil surface. Different incident angles were used for the different soil types to maximize Rayleigh waves propagating along the soil surface [43]. The incident angles used in this study are summarized in Table 1 and discussed further in detail in Section 3.1. An acoustic barrier was placed at 0.1 m in front of the ultrasonic receiver and on the soil surface, and the dimensions of which were 0.05 m (length) × 0.1 m (width) × 0.05 m (height) with evenly spaced four-panel walls (see Figure 2a). Note that the acoustic barrier was placed onto the surface of the specimens although the other experimental configuration was fully contactless. Therefore, the lift-off distance was maintained at 0.005 m throughout testing while the acoustic barrier was placed onto the surface.

To explore the relationships between soil moisture and Rayleigh waves, the prepared soil specimens were saturated and then dried in a temperature–humidity-controlled chamber during experiments. There might be unintended and undiscovered drying effects on Rayleigh waves to analyze soil moisture due to the hysteretic effects of water filling and drying the soil. However, for the sake of experimental simplicity, we assumed that these effects were relatively negligible compared to the impacts of soil moisture on the leaky Rayleigh waves. A drying cake method was used to reduce the friction between the soil and the acrylic box, which entailed coating the four walls and bottom of the box with a thin layer of Vaseline [44]. In addition, the chamber was maintained at the constant temperature and humidity of 35${\;}^{\circ }C$ and 40%, respectively, to minimize their impacts on acoustic waves. Soil moisture was measured using TEROS 12, inserted into an identical and independent specimen. The accuracy and resolution of the soil moisture sensor were 0.03 m${}^{3}$ m${}^{-3}$ and 0.001 m${}^{3}$ m${}^{-3}$, respectively. The soil moisture and leaky Rayleigh wave were measured every 5 min until the soil fell to the nearby residual soil moisture.

### 2.4. Random Forests

In this study, we used random forest regression due to its versatility to estimate soil moisture with the different dynamic parameters of leaky Rayleigh waves measured on the surface. Instead of developing mechanistic models that could explain causal relationships between the soil moisture and leaky Rayleigh waves, we applied this machine learning-based approach to the waves to overcome the limited nature of previous research on this topic. In addition, the purpose of this study was not to explore their relationships but to explore the potential of leaky Rayleigh waves to estimate soil moisture without soil disturbances. The random forest used in this study was originally proposed by [45] and has been used to solve regression and classification problems. This machine learning technique is a non-parametric algorithm with an ensemble of uncorrelated decision trees that are used to regress and classify problems. In recent years, this machine learning technique has been widely implemented, including biogeochemistry (e.g., [46,47]), ecology (e.g., [48,49]), hydrology (e.g., [50,51,52]), and wave analysis (e.g., [53,54]).

Random forest regression utilizes the bootstrap aggregation (bagging) of regression trees. With this algorithm, *N* sub-datasets are sampled from the original dataset, which is trained with *N* sub-models. All the trained *N* models are combined into one final model [45]. The use of the random forest is straightforward and does not require high computational costs for training a model, compared with other machine learning algorithms [55]. However, a large number of trees could slow down the algorithm for real-time predictions. In this study, we used an ensemble of 500 regression trees using the bootstrap aggregation without replacements. The number of predictors sampled for splitting at each node was set to be 5. For the training strategy, we extracted the features of leaky Rayleigh waves measured on the surface while drying the soils. Dynamic parameters used in this study were normalized amplitude, energy, phase, and period of leaky Rayleigh waves, of which the first two were used to minimize bias on the training processes (see Section 3.4 for details).

## 3. Results

### 3.1. Critical Angle Experiment

**Figure 4:**
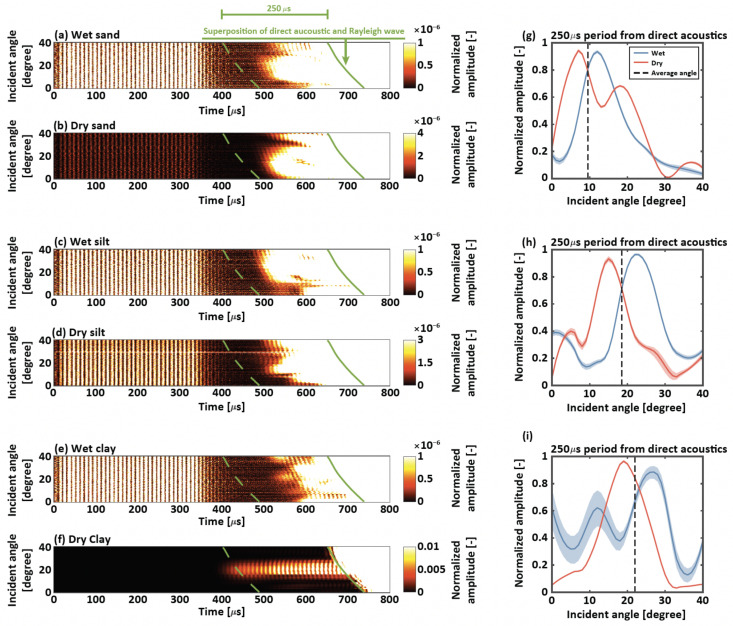
Critical angle experiments. Stacked and superposed ultrasonic signals from the 8 channels under different incident angles (y-axis) for (**a**) wet sand, (**b**) dry sand, (**c**) wet silt, (**d**) dry silt, (**e**) wet clay, and (**f**) dry clay. The hot colors in (**a**–**f**) represent normalized amplitudes. Here, separate data normalization was conducted under different soil and water conditions for visualization purpose. Averaged normalized amplitudes obtained during 250 μs from the direct acoustic arrivals are shown for (**g**) sand, (**h**) silt, and (**i**) clay. Red and blue lines represent wet and dry conditions, respectively. The shaded areas in (**g**–**f**) represent the 20th and 80th percentiles.

The excitations of the Rayleigh wave only require a free and viable surface, but only a joint half-space medium could generate an undistributed surface wave pulse [56]. This wave travels along the boundary between the air and the soil for the case of this study. At the critical angle, the maximum amplitude and energy of Rayleigh waves can be observed [43]. That is, the minimum wave penetration depth occurs at the critical angle by reducing the internal reflection of the acoustic waves. To determine the incident angle close to the critical angle to maximize leaky Rayleigh wave energy generations, we explored the impacts of different incident angles (ranging from 0 to 40 degrees) on the amplitude of the leaky Rayleigh waves under wet (fully saturated) and dry cases. Stacked and superposed ultrasonic signals from the eight channels under different soil types and incident angles and two levels of soil moisture are shown in Figure 4a–f. From a comparison between the wet and dry cases, the maximum amplitude under the dry soil occurred at the lower incident angle compared to that under the wet soil. An increase in soil air occupying the soil pores and the drying-induced increase in soil strength resulted in lower critical angles under the dry cases compared to the wet cases.

Which incident angle should be used for each soil type? To explore this question, we first identified the arrival time of the direct acoustics for the incident angle experiments (solid green lines in Figure 4a–f). We used the Akaike information criterion [57] to identify the arrival time of leaky Rayleigh wave. However, the arrival times of the direct acoustics were different for each receiver since the eight channels of the micro-electromechanical system were located at a distance of 5 mm. To overcome this issue, we used the earliest arrival time among identical incident angle experiments for different soil conditions. We then averaged normalized amplitudes obtained during 250 μs from the estimated direct acoustic arrival times (Figure 4g–i). The maximum normalized amplitude occured at the incident angle of 8${}^{\circ }$, 11${}^{\circ }$, 17${}^{\circ }$, 24${}^{\circ }$, 17${}^{\circ }$, and 27${}^{\circ }$ for dry sand, wet sand, dry silt, wet silt, dry clay, and wet clay, respectively. Since it was not practical to modify the incident angle during the experiments, we averaged the observed incident angles for each soil type. The incident angle of 9.5${}^{\circ }$, 18.5${}^{\circ }$, and 22${}^{\circ }$ for sand, silt, and clay, respectively, were used in this study to predict soil moisture based on leaky Rayleigh waves observed on the soil surface. Note that natural soils consisted of different compositions of sand, silt, and clay particles, leading to different critical angles. However, exploring their critical angles was beyond the scope of this paper.

### 3.2. Dynamic Parameters Dependent on Soil Moisture

**Figure 5:**
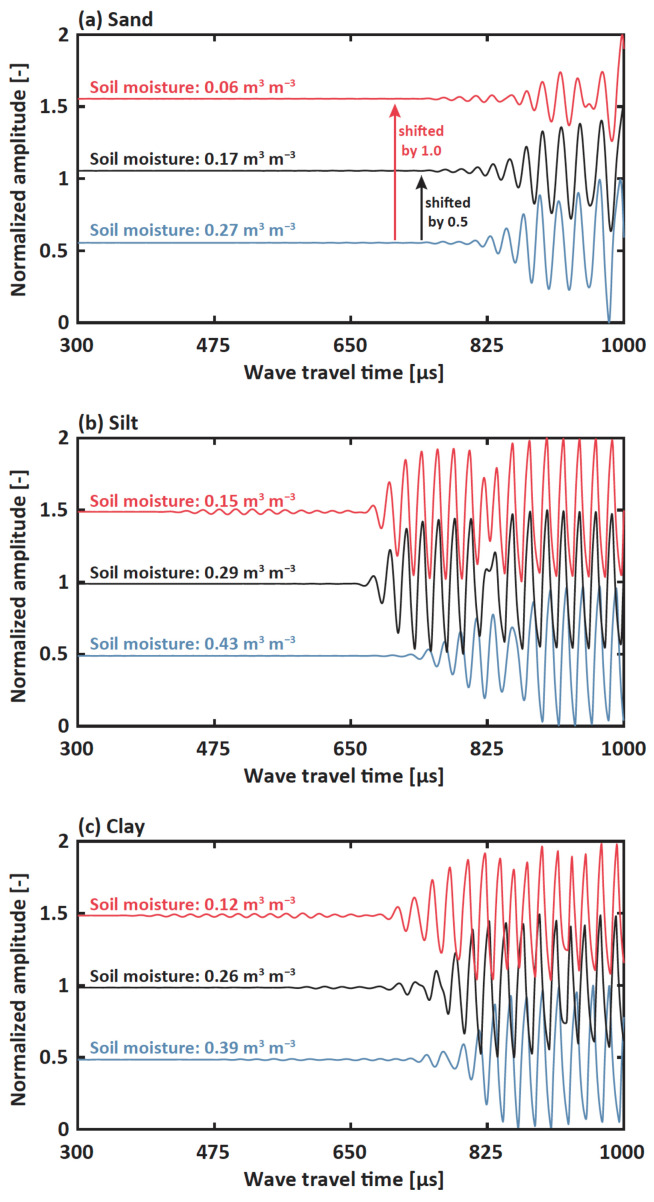
Obtained Waveforms for (**a**) sand (**b**) silt and (**c**) clay. Blue, black, and red colors represent nearly saturated, humid, and dry soil moisture, respectively, under different soil types. For visualization purposes, black and red lines were shifted by the normalized amplitude of 0.5 and 1.0, respectively.

The relation between soil moisture and dynamic parameters from obtained waveform was thoroughly evaluated, including time of flight (TOF), amplitude, energy, and phase. TOF is a fundamental characteristic of dynamic response and is easily identified from the experiment. Figure 5 presents the obtained waveforms over different soil moisture contents, where the waves were the superposition of leaky Rayleigh wave and direct acoustics. Since the soil hardness was dependent on soil moisture, the wavefront of the leaky Rayleigh waves was also influenced by soil moisture. Noted that the wavefront of the direct acoustics was not affected by soil moisture but influenced by travel distance through the acoustic barrier during the testing. As soil specimens became drier and their volume shrank into the acrylic mold, the direct acoustics were well disturbed by the acoustic barrier, where the travel distance became longer. In the case of leaky Rayleigh waves, the wavefront from both silt and clay specimens became faster as the soil moisture decreased (Figure 5b,c). However, the wavefront from sand showed different behaviors where the arrivals became slower as the specimen became drier (Figure 5a). We found that TOF was directly related to the hardness of materials during the process of evaporation. That is, the particles of silt and clay shrank resulting in decreased porosity and increased bulk density, leading to hardened soil conditions while drying in contrast to the case of sand particles, which did not stick to each other.

**Figure 6:**
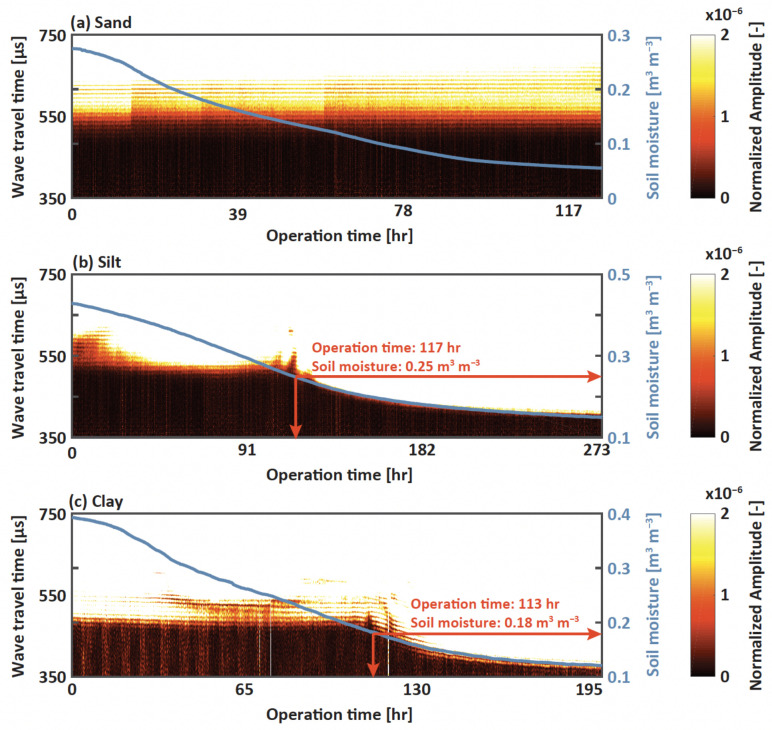
Stacked waveform images from (**a**) sand, (**b**) silt, and (**c**) clay. The bright colors in (**a**–**c**) represent normalized amplitudes. The x-axis represents the experimental operation time and left and right y-axes represent TOF and soil moisture, respectively.

The direct comparison between soil moisture and TOF is described in Figure 6, where the x-axis presents the operation time of experiments, and the left and right y-axes are TOF and soil moisture, respectively. A single waveform obtained every 5 min was stacked in a row, and the color of the image represented the normalized amplitude of waves. The wavefront was identified as a bright color. As the soil moisture continuously decreased during the experiments, the wavefront from silt and clay became faster at 117 and 113 h, and corresponding soil moisture levels were 0.25 and 0.18 m${}^{3}$ m${}^{-3}$, respectively (Figure 6b,c). The trend of the wavefront was similar to the decrease rate of soil moisture after certain periods; however, there were minimal variations in the wavefront when the moisture was relatively high. This unique trend implied that the TOF of leaky Rayleigh wave was partially correlated with soil hardness after a certain level of soil moisture. Noted that the TOF from the sand specimen was not related to decreases in soil moisture (Figure 6a). Instead, the arrivals slowed during the evaporation processes.

### 3.3. Wave Properties Dependent on Soil Moisture

**Figure 7:**
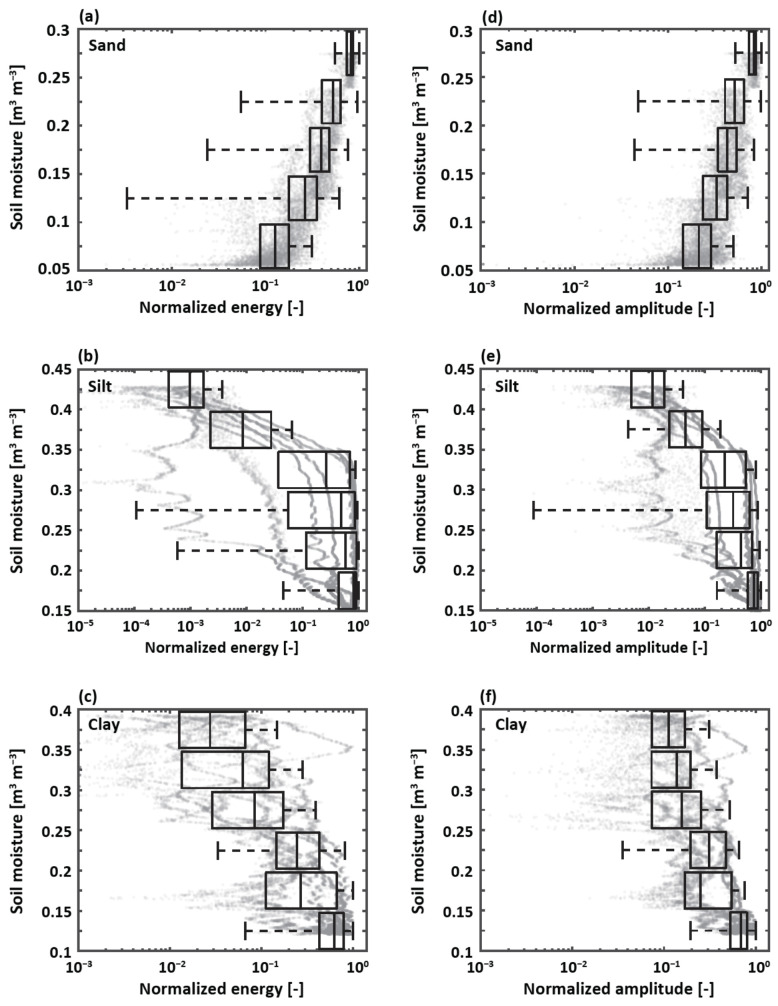
Box plots for wave properties with the soil moisture interval of 0.05 m${}^{3}$ m${}^{-3}$. The left column shows normalized energy for (**a**) sand, (**b**) silt, and (**c**) clay. The right column shows normalized amplitude for (**d**) sand, (**e**) silt, and (**f**) clay. The gray dots represent the estimated wave properties from the 8 receivers.

As discussed above, the TOF for leaky Rayleigh waves was not a reliable indicator to predict soil moisture. We instead explored the relationships between soil moisture and normalized energy, amplitude, phase, and period during the signal arrivals from 0 μs to 750 μs (Figure 6). These properties were analyzed with the soil moisture interval of 0.05 m${}^{3}$ m${}^{-3}$ to explore the potential of using leaky Rayleigh waves to estimate soil moisture. Strong relationships were determined between soil moisture and normalized energy and amplitude (Figure 7), but not between soil moisture and normalized phase and period (not shown). Noted that the wave dynamics for sand were different from those for silt and clay. For the case of sand, the magnitudes of normalized energy and amplitude decreased as the soil dried out, while the opposite was true for the cases of silt and clay. These findings can be explained by the soil plasticity in which a film of water covered the soil particles, allowing shape changes without rupture, especially for silt and clay. As the silt and clay became drier, they became rigid, allowing the soils to gain in strength, leading to increased wave energy and amplitude. In the case of sand, the coarse soil particles did not exhibit such soil plasticity and reduced soil strength during the drying processes due to the adsorption of water, which can act as a ‘glue’ between sand particles.

The distance between MEMS provided the different arrival times of leaky Rayleigh waves and direct acoustics and thus their unique characteristics. That is, the developed sensor array (eight MEMS channels) had the ability to provide imperative information on wave characteristics and properties to assess soil moisture, which could not be obtained by a single receiver. The analysis of leaky Rayleigh waves measured by a single receiver for monitoring soil moisture could lead to biased and misleading results as shown in Figure 7. In general, the normalized energy and amplitude of leaky Rayleigh waves obtained from the multiple channels provided comparable patterns dependent on soil moisture, but were not identical. However, we found that it was challenging to accurately estimate soil moisture merely based on these dynamic parameters.

### 3.4. Soil Moisture Prediction Using Random Forests

**Figure 8:**
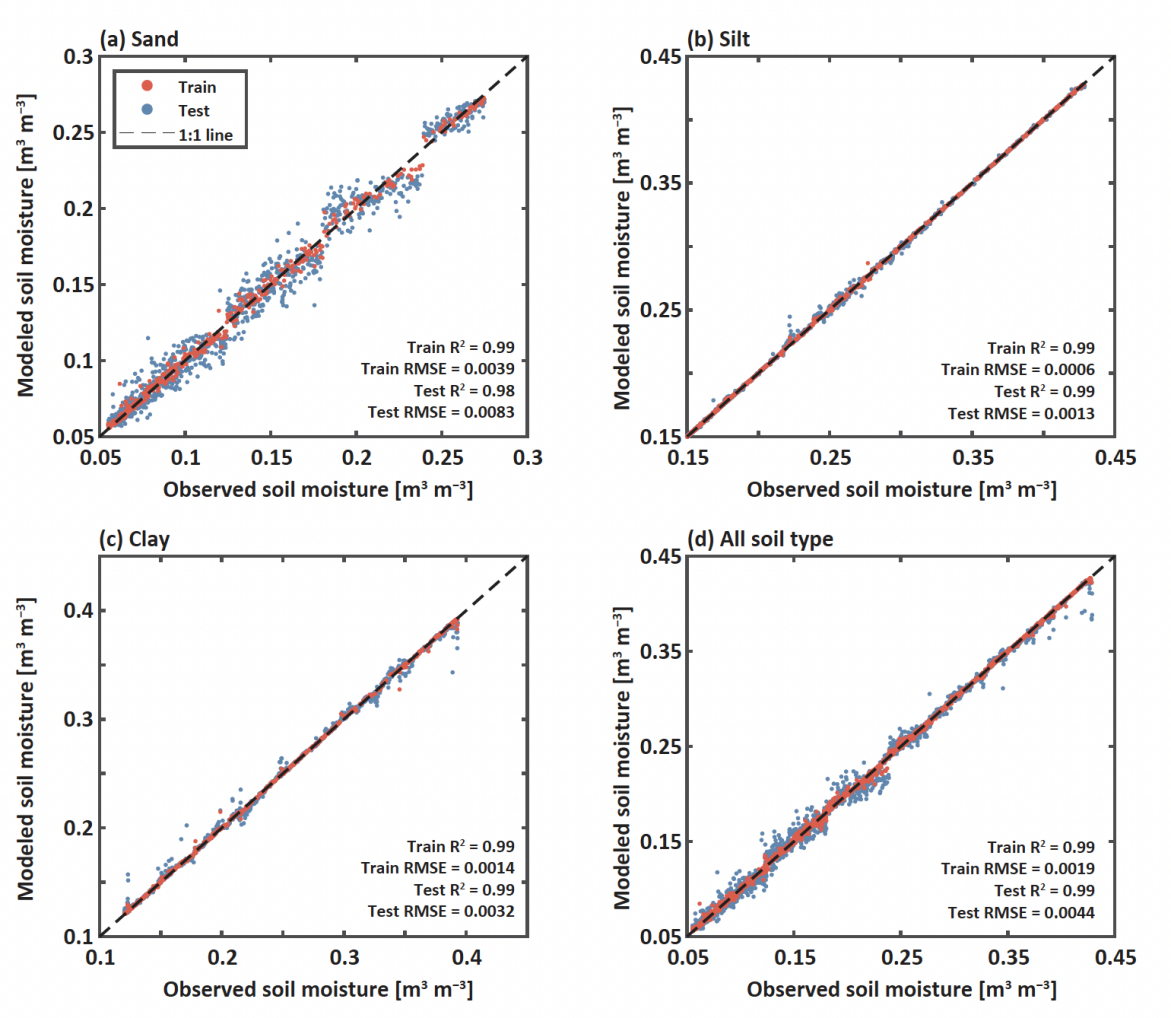
Model validation: 1:1 plot for the observed (x-axis) and predicted soil moisture (y-axis) using random forest for (**a**) sand, (**b**) silt, (**c**) clay, and (**d**) all soil types (a combination of sand, silt, and clay). The red and blue dots represent train and test cases, respectively. The dashed line represents a 1:1 ratio. A machine in the Intelligent Construction System Core-Support Center of Korea Basic Science Institute Center was partially used in this study.

To overcome the challenge of estimating soil moisture based on the dynamic parameters only, we applied random forest to the dynamic parameters and wave properties of leaky Rayleigh waves (Figure 8). We used the normalized energy and amplitude as inputs into the random forest to estimate soil moisture for sand, silt, and clay separately and in combination. To test and train the model, approximately 7200 samples were randomly selected and divided into 70% for training and 30% for testing. We found that leaky Rayleigh waves had excellent potential to estimate soil moisture without soil disturbances. The accuracy of predicted soil moisture based on the dynamic parameters and wave properties of leaky Rayleigh waves was exceptional for test data sets under all soil types (R${}^{2}$ ≥ 0.98, RMSE ≤ 0.0089). That is, the leaky Rayleigh waves applied to the soil surface contained the imperative information of soil moisture regardless of soil types.

## 4. Discussion

Soil moisture is a key element in controlling the exchange of water and energy between the land surface and the atmosphere and therefore terrestrial environments. There has been growing interest in the development of soil moisture sensors to improve underlying techniques and tools [58,59,60,61]. In particular, the need for preserving soil properties has been a great concern in monitoring soil moisture in recent years (e.g., [3,62,63,64]). In this respect, we developed a contactless ultrasonic system that could assess soil moisture without soil disturbances. The system was positioned on and applied to the soil surface. Three main soil types (sand, silt, and clay) were used to explore soil moisture dynamics based on leaky Rayleigh waves. We found high correlations between the energy and amplitude of leaky Rayleigh waves observed on the soil surface and soil moisture. Although we used a machine-learning based approach to extract soil moisture from leaky Rayleigh waves, the results found in this study could be regarded as the first step to devise mechanistic models in which key processes could be discovered. Our study indicated that leaky Rayleigh waves could be used as an indicator to contactlessly measure soil moisture, which might be regarded as analogous to an electric signal, which is the most widely used technique to measure soil moisture nowadays.

Although we conducted identical experiments in a controlled chamber, distinct wave patterns emerged from sand compared to silt and clay. For the case of sand, the relationships between the dynamic parameters and wave properties of leaky Rayleigh waves and soil moisture were relatively low, especially when compared with cases for silt and clay. During the experiments, we noted that the volumetric shrinkage enlarged gaps between the soils, especially for silt and clay but not for sand. To minimize the gaps, we applied Vaseline to the walls of the acrylic boxes to reduce friction between the soil and the boxes [44], leading to the volumetric shrinkages of silt and clay while drying without apparent gaps within the soils. That is, compacted silt and clay enhanced the sheer strength of silt and clay during evaporation processes, resulting in the increased energy and amplitude of leaky Rayleigh waves as opposed to the case of sand. In addition to the changes in the shrinkage of silt and clay, the degree of saturation is also related to changes in the Poisson ratio, pore pressures, and wave velocity, thus affecting leaky Rayleigh waves [65,66,67]. The combination of these dynamic phenomena could enable us to extract the features of soil moisture from leaky Rayleigh waves measured on the soil surface as demonstrated in this study.

One concern was that the dataset and experimental designs we used in this study were admittedly limited. Natural soils were inherently heterogeneous, leading to imperative but complex soil moisture dynamics (e.g., [68,69,70]). The controlled environments (constant temperature and humidity) used in this study were artificial ways to minimize the effects of uncertain variables that were not in the interest of this study. That is, the propagation characteristics of leaky Rayleigh waves on the medium’s surface were not only related to soil moisture, but also other factors, such as surface roughness, porosity, and temperature [71,72,73]. Since this study used a superficial wave, there was a limitation of effective depths to estimate soil moisture (a depth of approximately 5 cm). However, the contactless ultrasonic system that we developed in this study did not cause soil disturbances to assess soil moisture since the ultrasonic sender and receiver were positioned on the soil surface. In particular, we demonstrated that soil moisture could be continuously assessed based on the dynamic parameters and wave properties of the leaky Rayleigh waves without any soil contacts for the first time to the best of our knowledge. This contactless feature was a critical requirement to maintain the inherent soil structure and properties, and water-holding capacity for accurately monitoring soil moisture.

Beyond what we demonstrated in this study, three possible extensions related to the motion of the Rayleigh waves are listed as follows. First, the initiations of TOF variations should be further investigated for soil properties, such as wilting points. Certain time points were discovered from both silt and clay with different soil moisture contents. Therefore, the different soil types could be investigated to define the physical meaning of the initiations. Second, the shear modulus of soil could be estimated and evaluated through the dynamic response based on leaky Rayleigh waves. With the volume and weight of soil specimens during experiments, the shear modulus could be analyzed to develop a rheological model to explore their underlying mechanistic explanations. Noted that changes in the volume and weight were not critical parameters to estimate soil moisture in this study. Third, further experiments should be conducted to increase the effective depths of the proposed method by modifying the frequency of the ultrasonic waves. Fourth, the long-term fluctuation of soil moisture could be monitored with a fully automated system without soil disturbances. In this context, the proposed method could be further integrated with unmanned units in the field. With this integration, fully automated measurements could be achieved with minimal human intervention in soil environments.

## 5. Conclusions

This study demonstrated the potential of using leaky Rayleigh waves to continuously estimate surface soil moisture without any soil disturbances through the development of a contactless ultrasonic system. Based on the findings of this study, the following conclusions were drawn:

- The ultrasonic system developed in this study was able to clearly detect the changes in surface soil moisture through the excitation and reception of leaky Rayleigh waves.
- Strong relationships were identified between the energy and amplitude of leaky Rayleigh waves and soil moisture for the three tested soil types (sand, silt, and clay).
- The proposed deep learning approach showed the accurate and robust estimations of soil moisture using the dynamic parameters and wave properties of the observed leaky Rayleigh waves for the validation and test datasets, regardless of the soil texture and soil moisture contents (R${}^{2}$ ≥ 0.98, RMSE ≤ 0.0089 m${}^{3}$ m${}^{-3}$).

## Figures and Tables

**Table 1:** Specimen type and experimental setup.

Specimen Type	Particle Size	P-Wave Speed	Incident Angle	Porosity
Sand	2.0 to 0.05 mm	837∼1907 m s${}^{-1}$	9.5${}^{\circ }$	0.3
Silt	0.05 to 0.002 mm	528∼1552 m s${}^{-1}$	18.5${}^{\circ }$	0.42
Clay	Less than 0.002 mm	355∼1488 m s${}^{-1}$	22${}^{\circ }$	0.4

## Data Availability

The data presented in this study are available on request from the corresponding author.
